# Does the recent intensification of nationalistic and xenophobic attitudes in Eastern European countries adversely affect public mental health?

**DOI:** 10.1186/s12889-016-3785-3

**Published:** 2016-10-24

**Authors:** Andrzej Brodziak, Alicja Różyk-Myrta, Agnieszka Wolińska

**Affiliations:** 1 Institute of Occupational Medicine and Environmental Health, Koscielna 13, 41-200 Sosnowiec, Poland; 2 Institute of Nursing, University of Applied Sciences, Armi Krajowej 7, 48-300 Nysa, Poland

**Keywords:** Nationalistic attitude, Xenophobia, Xenophobic attitude, Eastern Europe, Public mental health, Positive psychology, Positive psychiatry, Social capital, Coherence, Immigration

## Abstract

**Background:**

The authors postulate that the recent intensification of the nationalist and xenophobic attitude in Poland and other Eastern European countries is detrimental to public mental health. The xenophobic attitude is accompanied by a higher incidence of anxiety and depression, disputes due to the polarization of opinions, a sense of embarrassment and a sense of contradictions with so-called Christian values, unfavorable demographic predictions and reduced life satisfaction.

**Discussion:**

The authors attempt to describe the sequence of adverse events that led to the intensification of xenophobia and characterize the current state of public mental health ***in European countries***. They formulate and propose possible actions which could counteract the consequences of that transformation.

**Short conclusion:**

The actions which may be undertaken to counteract the deterioration of public mental health can be based on the recommendations of so-called ‘positive psychology’ and ‘positive psychiatry’ as well as the principles of strengthening local social capital.

## Background

In 2015, after the massive influx of refugees to Europe and the change of the political option, the attitude of Polish society began to transform and become more nationalistic and xenophobic. Similar changes occurred in Hungary, the Czech Republic and Slovakia. These specific transformations are perceived and noted in other EU countries. Feedback causes further effects.

The question arises whether the described changes have an impact on public mental health [1–4] According to so-called ‘positive psychology’ [5] and ‘positive psychiatry’ [6], such transformations have an impact on the average state of health. A xenophobic attitude is accompanied by a higher incidence of anxiety, disputes by reason of the polarization of opinions, a sense of embarrassment and contradictions with so-called ‘Christian values’, adverse demographic perspectives, depression and reduced life satisfaction [7]. A x*enophobic attitude is usually the result of deeper social changes caused by economic crisis, loss of confidence in the existing political elite and an increase in populist argumentation*.

Therefore, we intend to discuss first the so-called ‘sequence of adverse events’ that led to the xenophobic attitudes, and then characterize the current state of public mental health and propose actions that could counteract the consequences of that transformation.

## Discussion

### The sequence of adverse events exacerbated the xenophobic attitude

Since the end of the 1980s, there has been an ideological dispute in Poland between two alternative camps in society. One of them is the vision of the society which is mono-ethnic, nationalistic, Catholic, conservative, as was formulated by Roman Dmowski, co-founder of the II Polish Republic. The second vision comes from the times of the creators of the Constitution of May 3, which assumes the existence of a multi-ethnic, multi–religious, civil state – a concept supplemented later by the liberal ideas of freedom, the market economy and democratic organization of the state. The violent ethnic events during the Second World War and the decisions of Stalin’s regime turned Poland into a monolithic state in terms of ethnicity. A quite similar transformation occurred in Czechoslovakia, divided later on into the more monolithic states of the Czech Republic and Slovakia, as well as the territorially truncated Hungary.

According to Timothy Snyder, the nationalist contemporary stance arises from the belief that prosperity and a just organization of society is most easily obtained by completely independent, fully sovereign national states [8]. He writes in a recent, very well-known article as follows:

I do not understand how people can interpret the interwar times as an object of nostalgia, and so this period is presented by nationalistic politicians promoting populism. It is a great illusion that then … nation states were durable and prospered well. It was the opposite. This period was short and it ended awfully… a nation is necessary but insufficient … history is established by greater entities, organisms, empires … If a government avoids any integration, it is wrong … There are two ways to deal with the effects of globalization. The first is to build a regional organism, which is like a roof over its head, which defends. The second way is the defense of national identity, focusing on the opposition, we - them…

Poland, the Czech Republic and Hungary are countries that did not participate in colonial conquests. Therefore, they did not bring to their territories larger groups of people of different racial, national and religious identity. The ways of living together in one country with citizens of different races, nationalities and religious beliefs are not known here from personal experiences of the last 70 years. Opinions on the possibility of co-existence and integration of so-called ‘strangers’ are based only on the opinions presented by publicists and politicians. As is known, the political and military events that occurred in the last few years in the countries of North Africa and the Middle East have led to the exodus of refugees, the emergence of the so-called Islamic State and acts of terror [9]. These events, of course, are widely commented on by Eastern European publicists and politicians. They also comment on the average beliefs and attitudes of Muslims and analyze in particular the possibility of integrating refugees in Western societies, especially the phenomenon of so-called ghettos.

*Many citizens of European countries consider that the administration of the EU and even present democratic national governments are not able to solve this new growing problem of the influx of refugees, and in particular to end the military conflicts generating this wave of migration*.

*The lack of a convincing design of solutions to these problems by both the leaders of the EU and democratic national governments tends to maintain the expected trend of nationalist and xenophobic attitudes*.

*Some observers of the social transformations in Europe postulate that the trend towards a more nationalistic and xenophobic policy in Europe started already with the economic crisis in 2008 (troubles of Greece and problems for Euro currency)* [10, 11]*. The refugee influx from 2015 only amplified this effect. This point of view can be justified. The citizens of many countries assume, in these circumstances, that the current ruling political elite is also responsible for the growing inequality, and high unemployment in some European countries* [12]*. For this reason the populist programs find approbation. Nevertheless, populist politicians always try to identify the guilty. Therefore a part of populist programs consists usually in accusing and prosecuting foreigners, newcomers*.

*It should be noted, however, that the xenophobic attitudes significantly increase always from the moment of the winning of a populist coup. The group of people who won in this way the power increases and consistently supports the nationalist and xenophobic sentiments*.

*It can be exemplified by the facts observed in Poland. Here the significance of the impact of the economic crisis of years 2008 – 2012 was not great, because our country as an exception during this period was in a good economic condition* [13, 14]*. The GDP growth was here permanently above 3 %, therefore our country was called “the green economic island”.*

*Whereas the spread of xenophobic attitudes worsen sharply after the victory of national and populist party. So it seems to us that the essential influence exerts the ideological attitude of the political elite.*

The intensification of nationalistic attitudes that have occurred recently in Western countries (Brexit, Marine Le Pen’s movement in France) have also had an impact on the convictions of citizens of Eastern European countries. The events and opinions recalled here are used for the current political struggle. As a result, the nationalist and xenophobic attitude is intensified.

### The arisen, contemporary, actual state of public mental health in Visegrad countries

There are centers carrying out objective assessments of the most important parameters, defining the state of public mental health in Eastern European countries. A very useful tool constitutes the annual report of the so-called ‘World Justice Project’ (rule of Law Index) [15]. This annual report provides a summary of indicators and graphic illustration of the situation in 102 countries of the world on objective findings concerning: 1. Compliance with the standards of democracy, 2. The level of corruption, 3. Transparency in governance, 4. The degree of respect for human rights, 6. Order and Security 7. The effectiveness of legislation, 8. Civil (social) justice, 9. Judiciary (criminal) justice. We propose looking at the graphs, for example, of Poland, the Czech Republic, Germany, Russia and Turkey, and to draw conclusions.

Among the parameters included in the ‘World Justice Project’ the features relating most to the problem of intensifying nationalistic and xenophobic attitudes are: freedom of expression, religious freedom, the right to privacy, the right to association and demonstration, labor rights, as well as the parameters for the senses of social and criminal justice. The new data, which will be announced after the observations made in 2016, may reflect changes which have occurred since the intensified inflow of refugees in the second half of 2015.

Another reliable source of data are the results of the project being run in Poland for years, known as Social Diagnosis [16].

*The project “Social Diagnosis” supplements indicators, which are presented annually by the Polish Central Statistical Office. The authors of this project derive data for their own questionnaires, which since 13 years are yearly distributed among 22 thousand people living in selected households. The collected data concern the conditions and quality of life of citizens as well as their attitudes, state of mind and behaviors.*

*There are taken into account all the important aspects of both economic and non-economic factors such as income, material affluence, savings, loans and indices of quality of education, medical care and ways to deal with problems, signs of stress, mental well-being, lifestyle, pathological behaviors, participation in culture, the use of modern communication technologies and many others.*

*The last available report is for the year 2015. The results of the survey conducted in 2015 are puzzling. Contrary to popular opinion expressed in casual conversations and most of publications in mass media - the authors of the project “Social Diagnosis” noted the improvements in a number of indicators relating to the conditions and quality of life. They write inter alia that the percentage of Poles who consider themselves as very or quite happy to be 81.5 %. This proportion increased by 3.1 % compared to 2013. The real income of households increased in comparison to 2013 by more than 12 % and the personal income by 10 %. There has been an increase in satisfaction in most aspects of life, especially in the evaluation of satisfaction with the situation in the country, the prospects for the future and the financial situation of the family. The economic stratification of Polish society diminished. Inequality of income distribution of households measured by the Gini coefficient falling in the last six years. The value of this ratio in 2009 was 0.318; in 2011 was 0.307; in 2013 was 0.305 and in 2015 was 0.285 (far below the average for the 27 countries of the European Union). Also the dissection of personal income fell from 0.373 in 2009 to 0.330 Gini coefficient in 2015. Income of the poorest households grew faster than the richest. The authors found that below the limit of extreme poverty in Poland in June 2015 lived 3.3 percent of households (1.8 % less than two years earlier, the least in the whole study period).*

*According to the authors of the project, there is in Poland little sign of development of civil society. Compared with previous studies, the percentage of people who trust other people increases from 12 % in 2013 to 15 per cent in 2015. Also slightly increased the sensibility to violations of the common good, but still almost half of the population is indifferent to acts of violating the public good.*

*However the indicators of social capital such as tolerance and tendency to form associations have not increased and even slightly declined being consistently low since the beginning of transformation and some of the lowest in Europe.*

*The above data (quoted here from the report counting 529 pages) seem to be paradoxical in light of the statement about current information related to the unfavorable state of mind of the nation in 2016, what is marked already in the title of this article.*

*Probably, the subjective explanation formulated by those who have a negative opinion on the national-populist upheaval, which happen in Poland will be reflected in the objective data that will provide the authors of the project “Social Diagnosis” in the report for 2016.*

It may turn out, however, that the mentioned parameters are not able to reflect the nature of new social trends. Many publicists emphasize that authoritarian populist politicians promise “to restore order, a sense of dignity, social discipline and to control the market and augment social support.” This brings to mind yet another possibility for estimating changes in mental health. Aaron Antonovski, in the times of relative social balance in the 1980s defined the concept of coherence and proposed to estimate such a state of the mind by means of his SOC-29 test [17]. Coherence is composed of: (1) the ability to understand events (comprehensibility), which allows one to perceive them as less stressful, (2) a sense of meaningfulness, consisting in the belief that it is worth being involved and creating one’s own life, (3) a sense of resourcefulness (manageability) [17].

The concept of coherence has become very well known. Hundreds of authors have published papers relating to the measurements of the level of coherence and proposed even a modification of its individual components [18]. The method of prevention and health promotion based on the concepts of coherence is called ‘salutogenesis’ [19].

It seems to us that the new, current research on the level of coherence in various social groups in different countries would bring important information to estimate the severity of the consequences of xenophobia.

*It should be noted that the sociological, practical problem is two-fold. It is necessary to consider the willingness and ability of immigrants to integrate in the society to which they came. In addition, there are variable degrees of difficulty to assimilate immigrants by the host communities.*

*Antonovsky’s concepts are highly relevant to the problem of xenophobia because he came from a family of immigrants and he was personally an immigrant, first in the United States, and then in Israel (an ‘immigrant’ country), where he also conducted extensive research on the relationship between well-being and health, depending on the degree of integration of newcomers* [19].

*He considered the so-called ‘pro-health quality of the receiving culture’.As the author of salutogenesis, he stresses that the receiving culture should promote conditions enabling citizens to experience life as comprehensible, manageable and meaningful. Comprehensibility arises from a stable culture that sends consistent messages to people, manageability is created by a culture that gives people the tools to live up to norms set by the culture, and meaningfulness is supported by a culture that values the role of people and gives them a place in the world. His original statement on this topic is as follows: “Clearly, if one has a high intelligence, lots of money, or a clear ego identity or lives in a stable, integrated culture – to mention some ‘generalized resistance resources’– there will be consequences not only for the emergence of a strong SOC, and therefore health, but for other areas of well-being as well”* [19].

*However, the results of contemporary research on changes in the level of coherence in populations manifesting a significant increase in xenophobic attitudes are not known.*

To assess the state of public mental health, it is worth yet quoting a recognized, widespread general definition of mental health. Manwell et al., promotes the formulation made by the Public Health Agency of Canada, which states that: “Mental health is the capacity of each and all of us to feel, think, and act in ways that enhance our ability to enjoy life and deal with the challenges we face. It is a positive sense of emotional and spiritual well-being that respects the importance of culture, equity, social justice, interconnections and personal dignity” [20].

In light of this definition, is easy to see that every citizen of Poland is troubled by the consequences of polarization of attitudes and views on the values which have been promoted during the past 25 years. Those with the convictions to being liberal, libertarian, tolerant are now in the defensive. It is difficult to achieve in this situation the determinants of good mental health, which require citizens to “enjoy life in the sense of emotional and spiritual well-being”. Yet other consequences of the intensification of the xenophobic attitude have occurred. The most obvious is the threat to public finances due to the slowdown of investment. A more subtle, but very unfavorable change consists in decreased “social capital”, i.e. the ability of citizens to cooperate with others [21–27]. Deepening the isolation of the country also diminishes the opportunities to improve the educational process and creativity.

Repealing from adopting even a very limited number of refugees is squandering a chance to force the development of professional, mental, cultural and linguistic skills of many institutions. The need to integrate people from different cultures is a challenge which stimulates different intellectual processes. The lack of implementing such efforts is detrimental to the development of practical skills of representatives of many professions.

### Possible remedies to counteract deterioration of public mental health

The question arises if there are any possible actions to mitigate the xenophobic attitude, even in very unfavorable conditions, when those ruling the country are not interested. It seems to us that such actions are possible. They result from the methods of ‘positive psychology’ and ‘positive psychiatry’ [5, 6], the principles of strengthening the local social capital [28–35] and the acquired knowledge of psychological regularities related to the formation of xenophobic attitudes, especially in children and adolescents [36–42]. These include: (1) specific publicist and cultural interventions (promotion of specific novels, movies) [43, 44] and (2) the promotion of certain already-developed software tools and social media e.g.: Viacharacter – a survey on character strength [45, 46], Sense of Coherence – Orientation to Life Questionnaire [47] or Learning to live together [48]. The efforts made in other European countries should be noted. An example is the recently announced program of implementations of new ways of intensifying the integration of refugees announced in Germany [49].

*Readers of this article may feel the need to be more aware of what the methods are resulting from so-called ‘positive psychology and ‘positive psychiatry’ and the principles of ‘strengthening the local social capital’. It is quite easy to make a review of these methods because one can indicate relevant publications available on-line* [43–47].

*Counteracting a xenophobic attitude boils down to eliminating prejudices. Citizens do away with prejudices when open-mindedness, the recognition of the rights of others to have a different opinion, appreciation of diversity, tolerance and the freedom to make choices become values for them. Hence, Hershberger, one of the authors representing the domain of ‘positive psychology’ proposes the use of such methods of personality transformations as ‘Three good things’, ‘The interpersonal sharing of good news’, ‘Sacrificing unnecessary effort and considering whether it is good enough’, ‘Realization the strengths and virtues of one’s own character’, ‘Learned optimism (not always, not everything)’* [46].

*‘Three Good Things’ intervention simply requires an individual to write down three positive occurrences that happened during the day every night for 1 week and for each occurrence an answer to the question of why the good thing happened. ‘Realizing the strengths and virtues of own character’ can be supported by utilizing the website Viacharacter* [45],

*An example of another approach of positive psychology can be the postulate of augmenting so-called ‘psychological capital’* [50–54]*. Psychological capital is defined as a higher-order core psychological construct and includes four states: psychological resources of self-efficacy, optimism, hope and resiliency. All these four factors can be measured and enhanced.*

*The targets of the enhancing self-efficacy, optimism, hope and resilience can be treated as tasks of supportive psychotherapy. We tried to explain in one of our previous papers, accessible on–line, how time–consuming behavioral psychotherapy can be supported by determining so-called ‘therapeutic tasks’* [43].

*It should be noted, however, that having tools that help to eliminate prejudices which are prevalent in the population affected by xenophobia is not tantamount to effective influence. Actions are impeded by the possibilities to reach the public, especially in the situation of the unfavorable attitude of the current government.*

*That is why it is so important to pay attention to methods of strengthening so-called social capital, in other words the ability of members of the population to establish personal contacts, cooperation and collaboration.*

*The key feature, essential for enhancement of social capital is the trust to other people. It should be noted that the xenophobic attitude lies precisely in the lack of trust in others. So, all the method of counteracting xenophobia through the strengthening of social capital consists of augmentation of the trust to others.*

*Strengthening social capital is easier when the country is organized on the basis of local administration autonomy and when many non–governmental organizations exist. In other words, it is easier to strengthen social capital when the civil society is under development* [55–58].

*The important factors enabling the leveling of xenophobic attitudes consist in appropriate changes in education and an appropriate cultural atmosphere* [58]*. Unfortunately, the populist regime interferes negatively in the organization of education and promotes its specific ‘cultural policy’.It follows that overcoming xenophobic attitudes is associated with social and even political struggles.*

*It seems, that* a sociological study carried out among 2.2 million Polish citizens who have recently emigrated could be fruitful, as well as research among people who live near the population of one million immigrants who came *to Poland* from the Ukraine.

It is important to promote the equal rights of men and women, and even a 50 % parity in different bodies, because the attitude of women is more moderate and pacifistic. Efforts should be made to widely disseminate newly acquired knowledge on the reverse relationship between the capacity for friendship and attitudes of tolerance and xenophobia [39–42] and the overwhelming influence of mothers (parents) on the xenophobic attitudes of their children [36–38]. Research on transnational identity is also useful [59–62]. The experiences from the integration of foreigners should be gathered in communities and schools of all types [63–66]. One should promote the training of people willing to undertake counseling and stimulate integration with foreigners [65].

It is advantageous to promote the effective learning of foreign languages and mobility of young people for work, education and research in foreign countries.

## Conclusions

1. Today there are tools to objectively determine whether the recent resurgence of nationalist and xenophobic attitudes in many countries of the world is evidenced in an objective manner, and whether it causes adverse consequences in the state of public mental health. The estimation of such changes can be made, for instance, by tracking changes that are recorded in the annual reports of the ‘World Justice Project’ (rule of Law Index) [10].
2. It is possible to formulate remedies to counteract the deterioration of public mental health.
3. Although the reasons for the intensification of nationalist and xenophobic attitudes are very complex, the counteractions for these changes in social consciousness can be underaken not only by enlightened publicists and politicians but also by people who are concerned about the state of public health. They can refer to the methods of so-called positive psychology and positive psychiatry and widely known famous and reputable cultural products (movies, novels, mass media performances).
4. Development and implementation of the methods and tools influencing the whole population, which counteract spreading xenophobia is a new challenge for the people who care about the state of public health.
